# Preclinical Safety Assessment of *Bacillus subtilis* BS50 for Probiotic and Food Applications

**DOI:** 10.3390/microorganisms10051038

**Published:** 2022-05-17

**Authors:** Laura M. Brutscher, Claudia Borgmeier, Sean M. Garvey, Jessica L. Spears

**Affiliations:** 1 BIO-CAT Microbials, LLC, Shakopee, MN 55379, USA; lbrutscher@bio-cat.com; 2 BRAIN Biotech AG, 64673 Zwingenberg, Germany; cb@brain-biotech.com; 3 BIO-CAT, Inc., Troy, VA 22974, USA; sgarvey@bio-cat.com

**Keywords:** *Bacillus subtilis* BS50, probiotics, safety

## Abstract

Despite the commercial rise of probiotics containing *Bacillaceae* spp., it remains important to assess the safety of each strain before clinical testing. Herein, we performed preclinical analyses to address the safety of *Bacillus subtilis* BS50. Using in silico analyses, we screened the 4.15 Mbp BS50 genome for genes encoding known *Bacillus* toxins, secondary metabolites, virulence factors, and antibiotic resistance. We also assessed the effects of BS50 lysates on the viability and permeability of cultured human intestinal epithelial cells (Caco-2). We found that the BS50 genome does not encode any known *Bacillus* toxins. The BS50 genome contains several gene clusters involved in the biosynthesis of secondary metabolites, but many of these antimicrobial metabolites (e.g., fengycin) are common to *Bacillus* spp. and may even confer health benefits related to gut microbiota health. BS50 was susceptible to seven of eight commonly prescribed antibiotics, and no antibiotic resistance genes were flanked by the complete mobile genetic elements that could enable a horizontal transfer. In cell culture, BS50 cell lysates did not diminish either Caco-2 viability or monolayer permeability. Altogether, BS50 exhibits a robust preclinical safety profile commensurate with commercial probiotic strains and likely poses no significant health risk to humans.

## 1. Introduction

*Bacillus subtilis* is a Gram-positive bacterium with a long history of use in molecular biology, industry, medicine, and fermented foods [1,2]. *Bacillus* strains are particularly useful for their ability to produce and secrete enzymes in mass and amenability to genetic manipulation. In the past two decades, many strains of *Bacillus* spp. have been used as human probiotics and direct-fed microbial for animal health. Probiotics are live microorganisms that, when administered in adequate amounts, confer a health benefit on the host [3]. Probiotics may provide health benefits such as supporting digestion, gastrointestinal (GI) health, immune health, beneficial resident gut microbes, and mood and stress response [4,5,6,7,8]. Some of the most commonly used probiotic strains include members of the *Lactobacillaceae* family (*Bacillota* phylum, formally known as *Firmicutes*), including the *Lactiplantibacillus*, *Lacticaseibacillus*, and *Lactobacillus* genera. Common probiotic strains also include *Bacillus spp.* and *Weizmannia coagulans* (formally *Bacillus coagulans*) strains from the *Bacillaceae* family of the *Bacillota* phylum and *Bifidobacterium* spp. from the *Actinomycetota* (formally *Actinobacteria*) phylum. 

*Bacillaceae* species are well-suited for probiotic applications because they can be manufactured as spores that persist without refrigeration and resist the acidic and high bile salt conditions that occur throughout the GI tract of humans and monogastric animals [9]. *Bacillus subtilis* (or *B. subtilis*), in particular, has a history of safe consumption across the globe. *B. subtilis* has been used in traditional fermented foods of many East Asian cultures for centuries, including the use of *B. subtilis* subsp. *natto* for commercial production of natto, a traditional Japanese dish containing fermented soybean [10]. *B. subtilis* strains have also been detected in Korean kimchi, Egyptian kishk, and other cultural adaptations of fermented soy, including miso and thua nao [11,12,13,14].

In addition to work utilizing in silico and in vitro studies, several animal toxicity studies have demonstrated the safety of *B. subtilis* for human use [15,16,17,18,19,20]. Clinical trials of *B. subtilis* and *W. coagulans* (formally *Bacillus coagulans*) strain supplementations have also shown safety and tolerance in humans, as well as digestive and GI health benefits in subjects with inflammatory bowel syndrome [21,22,23,24,25], dyspepsia [26,27], as well as individuals with or without mild symptoms of GI distress [28,29,30,31,32,33,34,35,36,37,38,39,40]. For example, the *B. subtilis* strain MB40 has been shown to be safe and support GI health in a randomized, double-blind, placebo-controlled trial of 100 healthy adults [17,35]. Additionally, *B. inaquosorum* DE111 supplementation has been shown to be safe in both adult and pediatric human subjects [41,42,43,44,45,46,47,48]. Altogether, these studies provide a large body of clinical evidence that *Bacillaceae* spp., including *B. subtilis*, are safe for human consumption. 

In this work, we performed preclinical studies to determine the safety of *B. subtilis* strain BS50 for probiotic applications. BS50 is a unique *Bacillus subtilis* strain that was isolated from soil and shows promise as a probiotic; preliminary assays indicate that BS50 exhibits enhanced heat tolerance and survivability in a simulated gastric model (unpublished data). To date, no known serious adverse effects have been reported from *B. subtilis* doses up to 10 billion colony-forming units (CFU)/day. At least five *B. subtilis* strains are the subject of “generally regarded as safe” (GRAS) dossiers, for which the Food and Drug Administration (FDA) has issued “no objection letters” for safe use in food [49,50,51,52,53]. Furthermore, the European Food Safety Authority (EFSA) maintains a qualified presumption of safety (QPS) list of biological agents that includes *B. subtilis*, which allows their use in food with no restrictions on age or exposure limit [54]. 

It is essential to assess the safety of each individual strain before clinical testing and safe use in dietary supplements, food, and beverages. Importantly, several *Bacillus* spp., including *B. cereus*, are capable of producing emetic toxins (e.g., cereulide), hemolytic and non-hemolytic enterotoxins, as well as cytotoxins (e.g., cytotoxin K), all of which can cause serious illness in humans and animals [55,56,57,58,59,60,61]. Another potential concern for probiotic strains is the presence of antibiotic resistance genes with flanking genetic sequences than can enable horizontal transfer to pathogenic bacteria in the GI tract [62,63,64,65,66,67]. To assess if *B. subtilis* BS50 poses any safety concerns to humans ahead of clinical testing, the BS50 genome was screened for genes encoding virulence factors, *Bacillus* toxins, and antibiotic resistance. We also performed in vitro antibiotic susceptibility tests and viability and permeability assays in human colon-derived Caco-2 cells.

## 2. Materials and Methods

### 2.1. Bacillus subtilis BS50 Isolation

*B. subtilis* BS50 (ATCC Accession No. PTA-127287, hereafter referred to as “BS50”) is a Gram-positive, spore-forming facultative bacterium that was isolated at BIO-CAT Microbials, LLC (Shakopee, MN, USA) from soil collected from Gallatin County, Montana, USA (collected on 4 July 2015). Isolation was performed by diluting the soil sample in Butterfield’s buffer and heating the sample up to 80 °C for 7 min to enrich for spore-forming bacteria. Serial dilutions of the sample were then plated on tryptic soy agar (TSA) plates that were incubated at 37 °C overnight. BS50 was a product of one of the resulting colonies. 

### 2.2. Genome Sequencing

Genomic DNA was isolated from tryptic soy broth (TSB) shake flask cultures using Genomic Tip 100/G (Qiagen, Hilden, Germany) in accordance with the manufacturer’s instructions. To obtain high purity DNA appropriate for sequencing, DNA was extracted via Genomic Clean and Concentrator columns (Zymo Research, Irvine, CA, USA) and afterward checked for quality and quantity using the deNovix dsDNA Broad Range fluorometric Assay (Wilmington, DE, USA). The sample was multiplexed and pooled with other libraries using SQK-LSK109 chemistry, and Native Barcode Extension packs EXP-NBD104 and EXP-NBD114 from Oxford Nanopore Technologies (Oxford, UK). All necessary clean-up steps were carried out using Clean NA magnetic beads for next-generation sequencing (Clean NA, Waddinxveen, Netherlands). Genome sequencing took place on MinIOn FlowCells FLO-MIN106D over 48–72 h (Oxford Nanopore Technologies, Oxford, UK). The full genome was assembled with Flye [68] using default settings. The BS50 genome comprises a single, circular contig 4,150,844 bp in length. No plasmids were detected. The BS50 genome has a GC content of 43.7%.

### 2.3. BS50 Taxonomic Classification via Multilocus Sequence Typing

Using BLAST+ command-line software [69], the nucleotide BLAST (BLASTn) algorithm [70] was used to identify nucleotide sequences in the BS50 genome and 20 other *Bacillus* genomes that aligned with six genes from the genome of *B. subtilis* subspecies *subtilis*, strain 168—one of the longest existing and most extensively studied strains of *B. subtilis* (type strain Marburg derived) [71,72]: *rpoB* (GeneID: 936335), *purH* (GeneID: 936053), *gyrA* (GeneID: 940002), *groEL* (GeneID: 938045), *polC* (GeneID: 939620), and *16S rRNA* (GeneID: 936895). These genes are standard “housekeeping” genes for *Bacillus* spp. and are commonly used for phylogenetic analysis of *Bacillus* spp. [73]. For each strain, the sequences aligning to these six genes were then concatenated into single nucleotide sequences (~19,616 nt). The strains used for comparison were selected based on having a complete genome available in the NCBI (National Center for Biotechnology Information) database or if they were currently used in probiotic supplements or food (i.e., MB40, BEST195, and DE111). 

Multiple sequence alignment of the concatenated sequences for each *Bacillus* strain was performed using MAFFT [74] (accessed 10 June 2021). The multiple sequence alignment file produced by MAFFT was then input into MEGA X [75] for phylogenetic tree construction. Their evolutionary history was inferred using the Maximum Likelihood method and Tamura-Nei model [76]. Data was bootstrapped 50 times. The tree with the highest log likelihood (−30362.06) was chosen. Initial tree(s) for the heuristic search were obtained automatically by applying neighbor-joining, and BioNJ algorithms to a matrix of pairwise distances estimated using the Tamura–Nei model and then selecting the topology with a superior log-likelihood value. This analysis involved 21 nucleotide sequences. Codon positions included were 1st + 2nd + 3rd+Noncoding. There was a total of 15,093 positions in the final dataset. 

In order to further characterize the sequence identities between the whole genomes of BS50 and 20 other *B. subtilis* strains, pairwise BLASTn alignments between BS50 and each *Bacillus* strain were performed via the NCBI website (accessed 25 January 2022) by uploading BS50 as the query and the other *Bacillus* genome as the subject. Default settings were used.

### 2.4. BLASTn Screen for Known Bacillus Toxins

A BLASTn search was completed via the NCBI website (accessed 2 June 2021) to determine the presence or absence of toxin genes commonly associated with the *Bacillus* genus. A table of the genes that were screened is shown in Table 1. In addition, positive control genes were identified in *B. subtilis glutamyl-tRNA(Gln) amidotransferase subunit* and *B. cereus methionyl-tRNA synthetase*. These genes were used as a query against the subject sequence *B. subtilis* BS50 genome to demonstrate the BLASTn algorithm was able to generate a match both within and across species when one existed. Each toxin gene DNA sequence was identified using NCBI gene or NCBI nucleotide databases. The sequence for the *B. cereus* cereulide gene cluster (*cesHPTABCD*) was obtained from the 270 kb plasmid pCER270 sequence (NC_010924.1, location: 15094 to 38668) [77,78]. Finally, each toxin gene DNA sequence was used as a query against the subject sequence BS50 genome. All nucleotide BLASTn alignments were run using default parameters.

### 2.5. BLASTx Screen for Known Bacillus Toxins

A translated nucleotide BLAST search was completed via the NCBI website (accessed 2 June 2021) to determine the presence or absence of coding sequences that are homologous to toxins commonly associated with the *Bacillus* genus. Protein sequences related to the control and toxin genes previously included in the BLASTn analysis were identified (http://www.ncbi.nlm.nih.gov/protein (accessed on 4 June 2021). These protein sequences were used as subjects against the query *B. subtilis* BS50 translated genome. All BLASTx alignments were run using default parameters.

### 2.6. In Silico PCR Amplification of BS50 for Bacillus Toxins

In silico PCR amplification was accessed online (4 June 2021) to search the *B. subtilis* BS50 genome for toxins via gene primer matches [79]. Ten sets of sequence primers for *Bacillus* toxin DNA amplification [80,81,82] were used to complete the virtual PCR (Supplemental Table S2). The following parameters were used to closely mimic an actual PCR run: two mismatches allowed, no mismatch allowed in the last nucleotide of the 3′ end, and a maximum band length of 10,000 nucleotides. As a positive control for the primers, the same set of primers was screened against the *B. cereus* genome, generating matches in all cases. As a control for the virtual PCR protocol, primers for 16S rRNA were used to show that the program would find a match when one was present.

### 2.7. Secondary Metabolite Screen via AntiSMASH

To determine if BS50 has the capacity to produce secondary metabolites, the BS50 genome was submitted to the online database antiSMASH bacterial version 6.0.1 (accessed 18 January 2022) [83]. Default settings were used; detection strictness was set to relaxed, and the features KnownClusterBlast, ActiveSiteFinder, RREFinder, and SubClusterBlast were turned on.

### 2.8. Secreted Protein via SignalP 6.0 Analysis

To determine if the BS50 genome encodes secreted proteins, it was uploaded onto the online server PATRIC [84] and annotated and translated via the RAST Tool Kit (RASTtk) [85] (accessed 26 March 2021). The translated amino acid sequences from the annotated BS50 genome were then analyzed for the presence of secreted proteins using the online SignalP 6.0 database [86] by setting the organism as “other” and setting model mode to “fast”. SignalP 6.0 was accessed on 18 January 2022. SignalP utilizes a machine learning model that predicts the presence of signal peptide motifs (i.e., Sec/SPI, Sec/SPII, Sec/SPIII, Tat/SPI, Tat/SPII) and the location of their cleavage sites [86]. 

### 2.9. Virulence Factor Screen via VFDB

To assess if the BS50 genome encodes for virulence factors (VF) or proteins involved in VF synthesis, the virulence factor database (VFDB) [87] was accessed online (17 January 2022), and the “full dataset” of VF-associated protein sequences was downloaded. The “full dataset” includes 1381 amino acid sequences for both verified and predicted VF-associated proteins from 954 medically relevant bacterial strains, whereas the “core dataset” only includes sequences of experimentally verified VF-associated proteins. The full dataset includes 36 VF-associated proteins from 164 strains and eight species of *Bacillus*, including proteins related to adherence (e.g., BslA), antiphagocytosis (e.g., capsule), iron acquisition, enzymes (e.g., InhA), regulation (e.g., AtxA), secretion systems (e.g., T7SS), and toxins (e.g., ALO, anthrax toxin, cereulide, certhrax, CytK, HBL, and Nhe) [87]. Since the dataset was primarily curated from medically relevant *Bacillus* strains, VF detection in BS50 was potentially limited. Using the BLASTx algorithm [70] with local BLAST+ command-line software [69], the BS50 genome was translated and screened against the VF dataset. Hits with <20% coverage were excluded from analysis, and multiple hits aligned to the same region of the BS50 genome were screened for the hit with the highest bit score. 

### 2.10. Antimicrobial Resistance Gene and Mobile Genetic Element Screen

The BS50 genome was screened for antibiotic resistance factors using the Resistance Gene Identifier (RGI), which is part of the Comprehensive Antibiotic Resistance Database (CARD) [88,89]. RGI is a web-based platform that utilizes BLAST to predict complete “resistomes” from genomic and metagenomic data. The BS50 genome sequence was submitted to the RGI CARD webserver (accessed 24 April 2021) using the following criteria: Perfect, Strict, complete genes only, 95% identity nudge used. Identity nudge allows any loose hit with at least 95% identity to be scored as a strict hit. 

To screen the BS50 genome for mobile genetic elements (MGE), the “A CLAssification of Mobile genetic Elements” (ACLAME) [90] database, version 0.4, was downloaded (1 June 2021) and aligned against the BS50 genome using the BLASTn [70] command with local BLAST+ software [69] under default parameters. The database contains 125,190 nucleotide sequences of predicted MGEs from prophages, virus, and bacterial plasmids. The BS50 genome was screened for known insertion sequences using the online program ISfinder [91] (accessed 1 June 2021), which utilizes the BLASTn algorithm [70] to search for nucleotide sequences that match insertion sequences.

To assess if MGEs or insertion sequences present within the BS50 could play a role in antibiotic resistance gene transfer, the loci of the sequences were manually compared to the loci of antibiotic resistance genes. Mobile genetic elements and insertion sequences that were not within five Kb of the loci of antibiotic resistance genes were not considered to play a role in antibiotic resistance gene transfer [92].

### 2.11. Antibiotic Minimum Inhibitory Concentration (MIC) Evaluation of BS50

MIC evaluation of BS50 against eight commonly prescribed antibiotics (i.e., chloramphenicol, clindamycin, erythromycin, gentamicin, kanamycin, streptomycin, oxytetracycline, and vancomycin) was completed by BioSciences (Bozeman, MT, USA; report number 2105336-202). The MIC of each antibiotic was determined based upon the methodology described in Clinical and Laboratory Sciences Institute (CLSI) Document M07 11th edition [93]. BS50 cells (3.93 × 10^6^ CFU/mL per well) were exposed to each of the 10 different dilutions of each antibiotic in sterile nutrient broth. Following an appropriate incubation period, the MIC of each antibiotic was determined visually and documented. *Enterococcus faecalis* (ATCC Accession No. 29212) and *Staphylococcus aureus* (ATCC #29213) (2.96 × 10^6^ and 8.25 × 10^5^ CFU/mL per well, respectively) were tested in tandem with BS50 to verify the methodology performed in this study, and they exhibited MICs within the CLSI quality control range. BS50 was deemed susceptible or resistant to particular antibiotics based on specific MIC thresholds established by the European Food Safety Authority (EFSA) for *Bacillus* strains [94,95].

### 2.12. Blood Hemolysis Assay

BS50 was streaked onto sheep blood agar plates to assess its ability to lyse blood cells. After incubation overnight, the agar was inspected for alpha-or beta-hemolysis. Alpha-hemolysis, or incomplete hemolysis, is indicated by a discolored, darkened, or green medium color after test culture growth. Beta-hemolysis, or complete hemolysis, is indicated by a clear and colorless medium after growth. An indiscernible change in the color of the agar indicates that no hemolysis occurred (i.e., gamma-hemolysis).

### 2.13. Caco-2 Cell Viability Assay

The effects of BS50 cell lysate on Caco-2 cell viability were tested at Charles River Laboratories (Bristol, UK). Caco-2 cells are an immortalized epithelial cell line of human colorectal adenocarcinoma cells. To generate the cell lysate, BS50 cells were harvested from overnight bacterial cultures and washed. The cells were lysed via enzymatic and mechanical bead-based processes. The final lysate was filtered through a 0.2 μM filter to remove any remaining cells. The final sterile-filtered lysate was plated on TSA to ensure it was free of viable cells. A “blank” sample was used as a process control sample for the lysate production method. The blank sample was sterile, uninoculated media that was treated exactly as the lysates were, including all spins, washes, lysing, and filtering steps. To perform the assay, Caco-2 cells were harvested, counted, and plated into 96-well flat-bottomed plates at 1 × 10^4^ cells/well in 100 µL volumes and left to adhere overnight at 37 °C, 5% CO_2_ in a humidified chamber. Cells were treated with BS50 lysate and incubated for an additional 48 h. Controls included cells that were left untreated and cells that were fully lysed at the time of treatment. Cell treatments were done in technical triplicate. Caco-2 cell viability was assayed using a CellTiter-Glo^®^ intracellular ATP quantification assay (Promega, Madison, WI, USA), alongside an ATP standard curve as per the manufacturer’s guidelines. Luminescence was quantified using a GloMax^®^ Plate reader (Promega). Levels of intracellular ATP in test conditions were quantified using the standard curve. ATP concentrations were tested for statistical significance using the Kruskal–Wallis test followed by a post-hoc Dunn’s test with Bonferroni correction for multiple testing in R Studio (Version 4.0.5). *p*-values less than 0.05 were considered significant.

### 2.14. Caco-2 Cell Transepithelial Electrical Resistance (TEER) Assay

The TEER assay was used to determine the effect of BS50 on gut barrier permeability (Charles River Laboratories, Portishead, UK). To generate a Caco-2 monolayer, Caco-2 cells were seeded on Transwell inserts over 14 days. At day 14, the polarized Caco-2 monolayers were pre-treated with a 1:5 dilution of BS50 lysate, sterile media process control, or lipopolysaccharide (LPS) control and left to incubate for 48 h. There was also a non-treatment control. TEER was measured before treatment (0 h), and at 2, 4, 6, 24, and 48 h after treatment. The TEER assays were performed twice on separate dates, with separate cell lysate preparations. Since the starting TEER values (ohm/cm^2^) at 0 h varied across treatments and trials, the TEER fold-changes were calculated relative to 0 h. Fold-change data from both trials were then combined and statistically analyzed as duplicates via the Kruskal–Wallis test, followed by a post-hoc Dunn’s test with Bonferroni correction for multiple testing in R Studio (Version 4.0.5). *p*-values less than 0.05 were considered significant. 

## 3. Results

### 3.1. Taxonomic Classification of BS50

**Figure 1:**
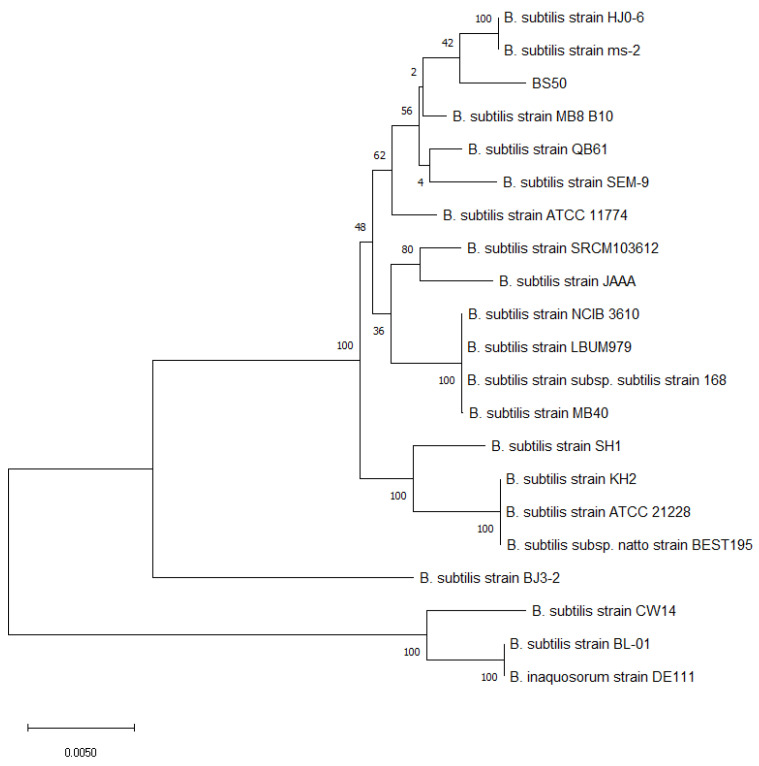
Maximum likelihood phylogenetic tree of BS50 and 20 other *B. subtilis* strains based on a concatenated sequence of the genes *rpoB*, *purH*, *gyrA*, *groEL*, *polC*, and 16S rRNA (15,093 nt). The bar indicates the rate of substitutions per nucleotide.

To confirm that BS50 is taxonomically a *Bacillus subtilis* strain, a phylogenetic tree of BS50 and 20 *Bacillus* strains was generated using concatenated ~20,000 nt sequences containing six “housekeeping” genes (i.e., rpoB, purH, gyrA, groEL, polC, 16S rRNA) [73]. The phylogenetic tree shows that BS50 aligns closely with other common *B. subtilis* strains, including the *B. subtilis* type strain 168 and *B. subtilis* MB40, a commercial probiotic strain (Figure 1). BS50 also closely aligns with commercial stains previously classified as *Bacillus subtilis* subsp. such as *B. inaquosorum* DE111. Pairwise whole genome alignments were performed between BS50 and the other *Bacillus* strains using BLASTn (Supplemental Table S1). Bacterial genomes sharing at least 95% average nucleotide identity are generally accepted as belonging to the same species [96,97]. The BS50 genome has 98.5% sequence identity to *B. subtilis* MB40 and 99.0% identity to *B. subtilis* subsp. *natto* BEST195, a *B. subtilis* strain commonly found in Japanese fermented natto beans (Supplemental Table S1). These data further support the classification of BS50 as a bona fide *B. subtilis* strain. 

### 3.2. BLASTn Screen for Known Bacillus Toxins

To screen the BS50 genome for toxin-encoding genes, the nucleotide sequences of known *Bacillus* toxins were aligned against the BS50 genome using BLASTn. The control genes, *gatA* and *metG*, yielded positive matches of 98% identity with 100% sequence coverage and 71% identity with 95% sequence coverage, respectively. The *metG* gene from *B. cereus* was used as a control for cross-species sequence matches to ensure that BLASTn could identify matches within BS50 when a gene from a different species was used as the input. Because *B. subtilis* and *B. cereus* are different species, a high identity is not expected. Thus, 71% identity with 95% sequence coverage satisfies its use as a control gene for cross-species matches (Table 1). No significant similarities were found between the query toxin sequences and the BS50 genome. The identified matches, including *HblA*, *entFM*, *cytK*, and *NheA*, *B*, *C* from *B. cereus* and *NheA*, *B*, *C* from *B. mycoides*, were the only partial matches that covered less than 25% of the toxin gene sequences. 

The *B*. *cereus* cereulide gene cluster (*cesHPTABCD*) from the 270 kb plasmid pCER270 sequence (NC_010924.1, location: 15094 to 38668) was also aligned against the BS50 genome. Only 50% coverage and 79% sequence identity were achieved, suggesting an incomplete cereulide gene cluster in the BS50 genome (Table 1).

### 3.3. BLASTx Screen for Known Bacillus Toxins

To further account for the ability of BS50 to produce toxin-encoding genes, the translated BS50 genome was aligned against the amino acid sequences of known *Bacillus* toxins using BLASTx.

The control proteins, GatA and MetG, yielded positive matches of 100% identity and 74.16% identity, respectively. Because *B. subtilis* and *B. cereus* are different species, a high identity was not expected, and thus, a 74.16% identity further satisfies its use as a control gene for cross-species matches (Table 2). 

No significant similarities were found between the query toxin protein sequences and the translated BS50 genome. The alignment between the translated BS50 genome and EntFM from *B. cereus* exhibited only 52.21% identity over a span of 113 amino acids. The EntFM protein sequence is 426 amino acids long, and the alignment only covered 26.5% of the EntFM protein sequence, which is insufficient coverage to conclude that BS50 produces the EntFM protein. The BS50 genome was translated and compared to the seven proteins encoded by the *B. cereus* cereulide gene cluster *cesHPTABCD*. There were matches between the BS50 genome and the protein sequences of CesA, CesB, CesC, CesH, CesP, and CesT all of which were less than 40% identical. CesH aligned at a locus of the BS50 genome that was roughly 1.3 Mb upstream of the other cereulide biosynthesis protein alignments. There were no significant matches with CesD (Table 2). 

### 3.4. In Silico PCR Amplification of BS50 for Bacillus Toxins

Virtual PCR only yielded matches using the positive control *16S rRNA* and *spoIVA* primers. None of the 11 queried toxin genes were detected in the BS50 genome using virtual PCR (Supplemental Table S2).

### 3.5. Secondary Metabolite Screen via AntiSMASH

To determine if BS50 has the ability to produce secondary metabolites, the BS50 genome was screened for secondary metabolite biosynthetic gene clusters using the online tool, antiSMASH [83]. Ten unique secondary metabolites (two terpene hits) were predicted in the BS50 genome (Table 3). 

### 3.6. Secreted Protein via SignalP 6.0 Analysis

To determine if the BS50 genome encodes for secreted proteins, the translated BS50 genome was analyzed for the presence of secreted proteins using the online SignalP 6.0 database [86]. As a result, 151 proteins were predicted with a greater than 50% likelihood to have Sec/SPI motifs, 93 proteins were expected to have Sec/SPII motifs, four proteins were predicted to have Tat/SPI motifs, and three proteins were predicted to have Sec/SPIII motifs.

### 3.7. Virulence Factor Screen via VFDB

To assess if BS50 genome encodes for virulence factors (VF), the virulence factor database (VFDB) [87] was aligned against the BS50 genome using BLASTx. There were 12 hits for VF-associated proteins in the BS50 genome (Table 4). 

### 3.8. Antibiotic Resistance Gene Analysis

The online tool RGI was used to screen the BS50 genome for antibiotic resistance genes. RGI identified one perfect, three strict, and 275 loose hits. Of the 275 loose hits, only 12 hits had at least a 95% identity and were nudged to strict hits (Table 5). Based on the presence of a gene with roughly 98% identity to *aadK*, an aminoglycoside 6-adenylyltransferase that is part of the ANT6 gene family, BS50 is predicted to be resistant to streptomycin. BS50 is also predicted to be resistant to the macrolides spiramycin and telithromycin due to the presence of *mph(K)*, a macrolide phosphotransferase. Additionally, BS50 is predicted to be resistant to tetracycline due to the presence of a tetracycline efflux pump (*Tet(L)*). In total, there are 16 potential resistance gene hits including *aadK*, *mphK*, and *tet (45)*, but only seven hits that cover more than 90% of the reference gene sequence. 

### 3.9. Insertion Sequences and Mobile Genetic Element Analysis

To assess if the antibiotic resistance genes present within the BS50 genome have the ability to be horizontally transferred to other bacteria, the BS50 genome was screened for insertion sequences using ISfinder and other mobile genetic elements using the ACLAME database (4.0). ISfinder found no matches between the BS50 genome and known insertion sequences with coverages greater than 15%. There were 122 unique loci in the BS50 genome that aligned with known mobile genetic element sequences from the ACLAME database with greater than 50% coverage, e-values less than 1.3 × 10^−11^, and bit scores greater than 65. To assess if these putative mobile genetic elements could play a role in antibiotic resistance gene transfer, the loci of sequences in the BS50 genome matching mobile genetic elements were then compared to the loci of antibiotic resistance genes identified via RGI. Out of the 122 loci that aligned to mobile genetic elements from the ACLAME database (4.0), one was found within five kb of an antibiotic resistance gene. The nucleotide sequence for the cupin domain-containing protein (NC_006322.1 (1,461,102 to 1,461,695)) was detected 1641 bp upstream of the blt-encoding gene (start position: 3,686,740; stop position 3,687,924). However, the nucleotide sequence for the cupin domain-containing protein only aligned to the BS50 genome with 80.3% similarity and 67% coverage, for which the 174 nt of the 5′ region did not align. 

### 3.10. MIC Evaluation of BS50 against Eight Antibiotics

BS50 sensitivity to eight medically relevant antibiotics, including chloramphenicol, clindamycin, erythromycin, gentamicin, kanamycin, streptomycin, oxytetracycline, and vancomycin was determined by MIC methods [93]. BS50 was susceptible to seven of eight antibiotics and exhibited resistance against streptomycin (Table 6). 

### 3.11. Blood Hemolysis Assay

To characterize any potential hemolytic activity, BS50 cells were streaked onto sheep blood agar plates and incubated overnight. The agar displayed a greenish hue surrounding the streaks where BS50 colonies grew, indicating that BS50 exhibits alpha-hemolysis.

### 3.12. Caco-2 Cell Viability Assay

**Figure 2:**
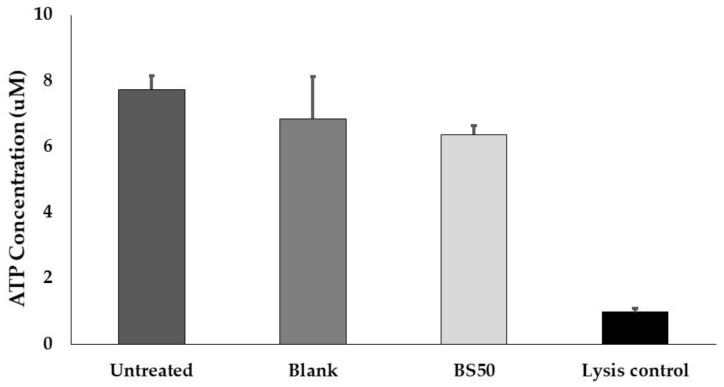
Effect of BS50 lysate treatment on Caco-2 cell viability after 48 h, as determined by ATP concentrations. Assay controls included untreated Caco-2 cells and cells that were fully lysed at the time of treatment. Data are expressed as mean ± standard deviation across technical replicates (*n* = 3).

Caco-2 cells were treated with BS50 lysate to test for deleterious effects on cell viability. While there was a significant difference in ATP concentrations between the cell lysis control and the untreated control (*p* = 0.014), the cells exposed to BS50 lysate showed similar ATP concentrations to the untreated control (*p* = 0.423) (Figure 2). Similarly, there was no significant difference in ATP concentrations between the untreated Caco-2 cell control and the blank sample, nor between the BS50 treatment and blank sample treatment. 

### 3.13. Caco-2 Cell TEER Assay

**Figure 3:**
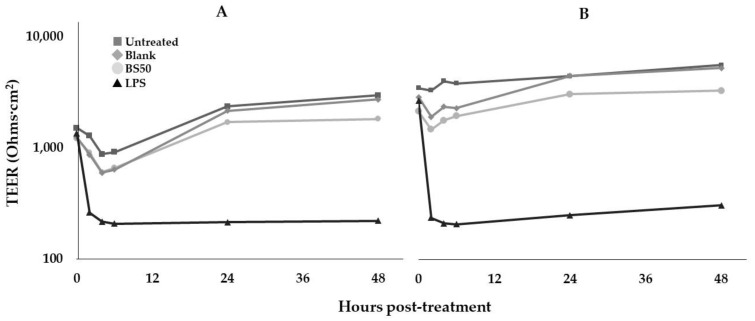
Effects of BS50 lysates on Caco-2 cell monolayer TEER in two separate trials (**A**,**B**). TEER was measured before treatment (0 h) and 2, 4, 6, 24, and 48 h after treatment. Square, untreated Caco-2 cells; diamond, “blank” lysate processing control; circle, BS50 lysate treatment; triangle, LPS treatment (TEER reduction control). Data are shown as two separate trials without replication within each trial (*n* = 1). Values on the *y*-axis are plotted on a logarithmic scale.

TEER assays were performed to determine the effect of BS50 on gut barrier permeability (Figure 3). Due to variations in the initial TEER measurements across wells, fold-changes relative to 0 h from both trials were combined into one data set for statistical analysis. There were no significant differences in TEER fold-change values between the untreated control, blank process control, and cells treated with BS50 lysate at both 24 h and 48 h post-treatment (*p* > 0.2), whereas the LPS control lowered TEER compared to all other treatments at 24 h (*p* < 0.006). 

## 4. Discussion

Spore-forming bacteria, particularly several *Bacillaceae* strains, are increasingly used in dietary supplements, food, and beverages due to their resistance to high temperatures and stability during manufacture, storage, and transportation [98]. Furthermore, the European Food Safety Authority (EFSA) has identified 17 *Bacillaceae* spp. with Qualified Presumption of Safety (QPS) status, including *B. subtilis*, *B. amyloliquefaciens*, *B. licheniformis*, *W. coagulans*, and *P. megaterium*, which are used as probiotics for humans and animals [54]. Regardless of the established safety of numerous *Bacillaceae* species, it is important to assess the safety of each individual strain, as reflected in the QPS qualifications that strains are required to meet (e.g., lack of acquired antimicrobial resistance, lack of cytotoxicity). We show here that *B. subtilis* strains BS50 show a robust preclinical safety profile. BS50 is a unique *B. subtilis* strain with at least 98% sequence similarity to commercial probiotic strains such as *B. subtilis* subsp. *natto* and *B. subtilis* MB40 (Supplemental Table S1). 

*Bacillaceae* spp., such as *B. anthracis*, *B. cereus*, and *B. thuringiensis*, are pathogenic in humans and animals [55,56,57,58,59]. *B. cereus* produces the emetic toxin cereulide, enterotoxins haemolysin BL (Hbl) and non-hemolytic enterotoxin (Nhe), and cytotoxin K (CytK) [60,61]. Other strains such as *B. subtilis*, *B. mojavensis*, *B. pumilus*, and *B. fusiformis* can produce cytotoxic and emetic toxins [99,100,101]. In order to address if BS50 is capable of producing toxins, we utilized BLASTn and BLASTx to screen the BS50 genome against the nucleotide and amino acid sequences of known *Bacillus* toxins, including the *Bacillus cereus* cereulide gene cluster (*cesHPTABCD*, 24-kb gene cluster belonging to the 270 kb plasmid pCER270) (Table 1 and Table 2). There were matches between the translated BS50 genome and the protein sequences of CesH, CesP, CesT, CesA, CesB, and CesC, but they had less than 40% sequence identity, and they did not contiguously align throughout the genome. Further, while most of these matches aligned with greater than 90% coverage, CesA and CesB aligned with less than 65% coverage, and there were no significant matches with CesD (Table 2). Given the absence of CesD in the BS50 genome, non-contiguous alignment, and the low sequence identity and/or coverage to CesH, CesP, CesT, CesA, CesB, and CesC, there is sufficient evidence to conclude that BS50 does not contain a functioning cereulide synthase cluster.

The BS50 genome was also screened in silico for virulence factors and secondary metabolites. It was found that the BS50 genome contains secondary metabolite biosynthetic gene clusters and encodes several proteins that are associated with virulence in pathogenic organisms. However, the products encoded by these genes are not innately toxic. Contrary to primary metabolites, secondary metabolites are non-essential small organic molecules that may contribute to evolutionary fitness over time, such as improving survival against competing organisms in the same niche [102]. For example, a few secondary metabolites (e.g., bacillibactin and fengycin) that are synthesized by non-ribosomal peptide synthases (NRPS) were predicted to be produced by BS50. Bacillibactin is a catecholate siderophore encoded by the *dhb* operon (as detected in Table 4) and is involved in the chelation and utilization of ferric iron [103,104].

Due to its ability to bind and remove iron, bacillibactin has been proposed to treat Parkinson’s disease since patients exhibit an accumulation of iron in the brain’s substantia nigra [105]. In silico analysis also predicted that BS50 produced fengycin, an established antimicrobial in preclinical studies and suggested bioactive in a clinical observational trial; The presence of fecal *Bacillus* spp. was correlated with the reduced fecal occurrence of the pathogen *Staphylococcus aureus* in a rural Thai population [106]. Preclinical experiments suggest that fengycin production by *B. subtilis* is required to exert this pathogen exclusion effect [106]. Two antibiotic-encoding genes were also detected in the BS50 genome, including bacilysin and bacillaene. Bacilysin is a non-ribosomally synthesized dipeptide antibiotic that inhibits Gram-negative foodborne pathogens [107,108,109]. Bacillaene is a polyene antibiotic that can accelerate biofilm formation and has activity against a broad spectrum of bacteria, including *S. aureus* and *E. coli* [110,111,112,113]. It functions by inhibiting bacterial protein synthesis, but it cannot inhibit eukaryotic protein synthesis. BS50 also encodes for genes involved in capsular polyglutamate synthesis and transport (i.e., *CapA*, *CapB*, and *CapC*). Polyglutamate can enhance the pathogenesis of *B. anthracis* and *S. epidermidis* by evading the innate immune response [114,115]. Interestingly, poly-γ-glutamic acid isolated from a novel *B. sonorensis* strain has been shown to inhibit *S. aureus* and *E. coli* growth [116]. 

Most of the secondary metabolites and VF-associated proteins that were detected in the BS50 genome are also widely present throughout many *Bacillus* genomes [102]. As mentioned in [102], surfactin, plipastatin/fengycin, bacillibactin, bacillaene, and bacilysin are produced by 99%, 97%, 99%, 77%, and 93% of *B. subtilis* strains tested. Subtilosin A is also produced by several *B. subtilis* strains, including Strain 22a, a wild strain of *B. subtilis* isolated from a fermented soybean product [117,118]. All four strains of *B. subtilis* and no other species isolated from the mushroom substrate (including *Lactococcus lactis*, *B. lichenimormis*, and *B. sonorensis*) produce subtilomycin [119]. As mentioned prior, BS50 encodes genes involved in the biosynthesis of polyglutamate (Table 4). Polyglutamate is produced by many commensal Bacillus strains and is found in several *Bacillus*-fermented foods, including natto [120]. In a study examining polyglutamate synthesis in fermented foods, 4.7%, 1.8%, and 3.0% of the *Bacillus*-like strains isolated from Cheongkukjang, Doenjang, and Kochujang samples, respectively, produced polyglutamate [121]. Because these metabolites/virulence factors predicted to be synthesized by *B. subtilis* BS50 are produced by other species of *B*. *subtilis*, these properties should be considered intrinsic. 

BS50 was also screened for the presence of antibiotic resistance encoding genes and susceptibility to antibiotics. The emergence of multidrug resistant pathogens is a major global health concern, and overuse of antibiotics has contributed to a greater incidence of antibiotic-resistant pathogens [62,122,123]. Additionally, antibiotic resistance genes present in plasmids, transposons, and integrons can be transferred from one bacteria to another via horizontal gene transfer [63,64,65,66,67]. The GI tracts of humans and animals contain complex and diverse microbial communities that may contribute to the transfer of antibiotic resistance genes from commensal organisms to potentially pathogenic bacteria [124]. BS50 was predicted to encode 16 antibiotic resistance genes that can provide resistance against multiple types of antibiotics, including fluoroquinolones, aminoglycosides, macrolides, lincosamides, tetracyclines, phenicols, nucleoside antibiotics, and peptide antibiotics (Table 5). BS50 was then tested in vitro for susceptibility/resistance against a comprehensive suite of medically relevant antibiotics as established by EFSA guidelines [94,95]; in vitro susceptibility tests determined that BS50 was resistant to the aminoglycoside streptomycin and susceptible to one phenicol antibiotic, two macrolides/lincosamides, two aminoglycosides, one glycopeptide, and oxytetracycline (Table 6).

Streptomycin resistance is widespread throughout *Bacillus* species and is most likely a part of their intrinsic genetic makeup rather than having acquired resistance from transferable genetic elements [125]. Regarding antibiotic resistance gene transfer, no plasmids were detected during BS50 genome assembly. While 122 regions of the BS50 genome aligned with mobile genetic elements from the ACLAME database, only one mobile genetic element was within five kb of any antibiotic resistance genes detected via CARD. The mobile genetic element cupin-domain-containing protein was detected 1641 bp upstream of the *blt* gene, which confers resistance against fluoroquinolone antibiotics and acridine dyes. However, 174 nt of the 5′ region of the sequence encoding for the cupin-domain-containing protein did not align to the BS50 genome, suggesting that this gene is non-functional and/or truncated. Thus, BS50 is at low risk of transferring antibiotic resistance genes to human gut-resident bacteria.

Of note, the BS50 genome encodes for a hemolysin, *putative membrane hydrolase* (*hlyIII*) (Table 4). In turn, BS50 was streaked onto sheep blood agar plates to assess its ability to lyse blood cells, and it was determined that BS50 exhibits incomplete hemolysis. Hemolytic activity has been detected throughout several *Bacillus* strains isolated from commercially available probiotics [126]. While this may present a safety concern if BS50 comes into contact with the bloodstream, the likelihood of an oral probiotic translocating through the intestinal barrier into the bloodstream is small and has only been reported at very low rates in hospitalized patients [127]. Nonetheless, to address potential concerns with gut barrier integrity and translocation, human colon-derived Caco-2 epithelial cell ATP viability and TEER tests were performed. We established that BS50 lysates did not negatively affect Caco-2 cell viability or monolayer permeability. Maintenance of Caco-2 cell viability and monolayer barrier integrity during BS50 lysate exposure, together with the in silico safety profile, suggest that BS50 will not be toxic to enterocytes in the human intestine or affect gut barrier integrity. A clinical trial in healthy adults has been initiated to better understand the safety and tolerability of BS50 in humans (ClinicalTrials.gov (last accessed on 18 April 2022). Identifier: NCT04655352).

## 5. Conclusions

Based on the results from in silico and in vitro analyses, BS50 is expected to be safe for human consumption. A clinical trial is being conducted to support the safe use of this strain by humans at anticipated rates of consumption from use in food or dietary supplements. 

## Figures and Tables

**Table 1:** Summary of BLASTn screening results for known *Bacillus* toxin genes in the BS50 genome.

Gene	Organism	Accession	Max Score	% Coverage	E-Value	% Identity
*gatA*	*B. subtilis*	938748	2405	100%	0	98%
*metG*	*B. cereus*	61578313	911	95%	0	71%
*HblA*	*B. licheniformis*	KM514479.1	No significant similarity			
*HblA*	*B. cereus*	KF681259.1	35.6	12%	0.021	82%
*HblC*	*B. cereus*	JQ039142.1	No significant similarity			
*HblD*	*B. cereus*	JQ039158.1	No significant similarity			
*NheA*,B,C	*B. cereus*	DQ885236.1	424	22%	9 × 10^−118^	70%
*NheA*,B,C	*B. mycoides*	DQ153260.1	82.8	3%	0.002	68%
*NheA*	*B. cereus*	FN825684.1	No significant similarity			
*NheA*,B,C	*B. thuringiensis*	EU925144.1	No significant similarity			
*entFM*	*B. cereus*	AY789084.1	59	14%	9 × 10^−09^	75%
*cytK*	*B. mycoides*	AY871809.1	No significant similarity			
*cytK*	*B. licheniformis*	KM657965.1	No significant similarity			
*cytK*	*B. cereus*	DQ019311.1	37.4	1%	0.044	92%
*HlyII*	*B. thuringiensis*	564444080	No significant similarity			
*cesHPTABCD*	*B. cereus*	NC_010924.1	109	50%	1 × 10^−22^	79%

**Table 2:** Summary of BLASTx screening results for known *Bacillus* toxin genes in the BS50 genome.

Protein	Organism	Accession	Max Score	E-Value	% Identity
GatA	*B. subtilis*	NP_388550.1	879	0	100%
MetG	*B. cereus*	WP_079994147.1	946	0	74.16%
cytK	*B. mycoides*	AAW56196.1	No significant similarity found		
EntFM	*B. cereus*	AAX14641.1	121	4 × 10^−29^	52.21%
cytK	*B. cereus*	AAY84864.1	No significant similarity found		
NheA	*B. mycoides*	AAZ82480.1	No significant similarity found		
NheB	*B. mycoides*	AAZ82481.1	No significant similarity found		
NheC	*B. mycoides*	AAZ82482.1	No significant similarity found		
NheA	*B. cereus*	ABI52601.1	No significant similarity found		
NheB	*B. cereus*	ABI52602.1	No significant similarity found		
NheC	*B. cereus*	ABI52603.1	No significant similarity found		
NheA	*B. cereus*	CBL95107.1	No significant similarity found		
NheA, partial	*B. thuringiensis*	ACM18211.1	No significant similarity found		
NheB	*B. thuringiensis*	ACM18212.1	No significant similarity found		
NheC, partial	*B. thuringiensis*	ACM18213.1	No significant similarity found		
HblD	*B. cereus*	AFN08801.1	No significant similarity found		
HblC	*B. cereus*	AFN08807.1	No significant similarity found		
HblA	*B. cereus*	AII31101.1	No significant similarity found		
HblD	*B. licheniformis*	AIR07774.1	No significant similarity found		
HblA	*B. licheniformis*	AIR07775.1	No significant similarity found		
cytK	*B. licheniformis*	AIS75096.1	No significant similarity found		
CesA	*B. cereus*	WP_002081542.1	1250	0	34.42%
CesB	*B. cereus*	WP_000953496.1	776	0	36.32%
CesC	*B. cereus*	WP_000590108.1	144	6 × 10^−38^	31.51%
CesD	*B. cereus*	WP_001008264.1	No significant similarity found		
CesH	*B. cereus*	WP_000291846.1	53	2 × 10^−07^	22.05%
CesP	*B. cereus*	WP_000680399.1	129	3 × 10^−33^	31.16%
CesT	*B. cereus*	WP_000764755.1	116	4 × 10^−29^	30.22%

**Table 3:** Summary of secondary metabolite screening results for BS50 using antiSMASH.

Cluster Type	Most Similar Cluster	% Identity
NRPS (Non-ribosomal peptide synthases)	Surfactin	78%
NRPS	Fengycin	100%
NRPS	Bacillibactin	100%
Other	Bacilysin	100%
Polyketide + NRP	Bacillaene	100%
RiPP: Thiopeptide	Subtilosin A	100%
RiPP: Thiopeptide	Subtilomycin	100%
CDPS	N/A	N/A
Terpene	N/A	N/A
T3PKS	N/A	N/A

**Table 4:** Summary of BS50 genome screening for virulence factors using VFDB.

Gene	Category	Organism	Accession	% Ident	% Coverage	E
*non-ribosomal peptide synthetase*, *DhbF*	Bacillibactin; Nutritional/ Metabolic factor	*B. sub* 168	NP_391076	99.1	99	0
*2*,*3-dihydroxybenzoate adenylase DhbE*	Bacillibactin; Nutritional/ Metabolic factor	*B. sub* 168	NP_389723	99.4	100	0
*isochorismate synthase DhbC*	Bacillibactin; Nutritional/ Metabolic factor	*B. sub* 168	NP_391078	98.5	100	0
*isochorismatase*, *DhbB*	Bacillibactin; Nutritional/ Metabolic factor	*B. sub* 168	NP_391471	99.7	100	0
*2*,*3-dihydroxybenzoate-2*,*3-* *dehydrogenase*, *DhbA*	Bacillibactin; Nutritional/ Metabolic factor	*B. sub* 168	NP_391079	99.2	100	0
*gamma-glutamyltranspeptidase*, required for polyglutamate anchoring to peptidoglycan	Capsule; Immune modulation	*B. sub* 168	NP_391469	98.9	100	0
*CapB*, involved in Poly-gamma-glutamate synthesis	Capsule; Immune modulation	*B. sub* 168	NP_391077	99.3	100	0
*CapA*, required for Poly-gamma-glutamate transport	Capsule; Immune modulation	*B. sub* 168	NP_391080	99.2	100	0
*CapC*, involved in Poly-gamma-glutamate synthesis	Capsule; Immune modulation	*B. sub* 168	NP_390062	100	100	0
*endopeptidase Clp* *ATP-binding chain* C	ClpC; Stress survival	*B. sub* 168	NP_391470	98.7	100	0
*(tufA) elongation factor Tu*	EF-Tu; Adherence	*Lm* EGD-e	NP_463763	72.6	89	0
(*hlyIII) putative membrane* *hydrolase*	Hemolysin III; Exotoxin	*Franc*.	WP_013922406	74.7	99	1.39 × 10^−142^

**Table 5:** Summary of antibiotic resistance genes detected in the BS50 genome using CARD.

ARO Term (Gene)	AMR Gene Family	Drug Class	% Identity	% Length	RGI Criteria
*ykkD*	small multidrug resistance (SMR) antibiotic efflux pump	aminoglycoside antibiotic, tetracycline antibiotic, phenicol antibiotic	100	101.9	Strict
*lmrB*	ATP-binding cassette (ABC) antibiotic efflux pump	lincosamide antibiotic	96.7	100.42	Strict
*ykkC*	small multidrug resistance (SMR) antibiotic efflux pump	aminoglycoside antibiotic, tetracycline antibiotic, phenicol antibiotic	100	100	Perfect
*tet*(*45*)	major facilitator superfamily (MFS) antibiotic efflux pump	tetracycline antibiotic	75.8	100	Strict
*mphK*	macrolide phosphotransferase (MPH)	macrolide antibiotic	97.7	100	Strict
*blt*	major facilitator superfamily (MFS) antibiotic efflux pump	fluoroquinolone antibiotic, acridine dye	99.8	98.5	Strict
*Bacillus subtilis pgsA with mutation conferring resistance to daptomycin*	daptomycin resistant pgsA	peptide antibiotic	99.7	90.53	Strict
*Bacillus subtilis mprF*	defensin resistant mprF	peptide antibiotic	99.7	76.87	Strict
*vmlR*	ABC-F ATP-binding cassette ribosomal protection protein	macrolide antibiotic, lincosamide antibiotic, streptogramin antibiotic, tetracycline antibiotic, oxazolidinone antibiotic, phenicol antibiotic, pleuromutilin antibiotic	98.5	75.5	Strict
*aadK*	ANT(6)	aminoglycoside antibiotic	97.8	63.03	Strict
*bmr*	major facilitator superfamily (MFS) antibiotic efflux pump	fluoroquinolone antibiotic, nucleoside antibiotic, acridine dye, phenicol antibiotic	100	47.3	Strict
*tmrB*	tunicamycin resistance protein	nucleoside antibiotic	97.6	42.13	Strict
*aadK*	ANT(6)	aminoglycoside antibiotic	97.2	39.44	Strict
*vmlR*	ABC-F ATP-binding cassette ribosomal protection protein	macrolide antibiotic, lincosamide antibiotic, streptogramin antibiotic, tetracycline antibiotic, oxazolidinone antibiotic, phenicol antibiotic, pleuromutilin antibiotic	96.4	27.24	Strict
*tmrB*	tunicamycin resistance protein	nucleoside antibiotic	100	26.9	Strict
*Bacillus subtilis mprF*	defensin resistant mprF	peptide antibiotic	100	16.36	Strict

**Table 6:** In vitro minimum inhibitory concentrations of antibiotics for BS50. The last column includes EFSA-recommended MIC thresholds for antibiotic resistance in *Bacillus* strains [94,95].

Antibiotics	Type	MIC (µg/mL)	EFSA MIC (µg/mL) Resistance Threshold
Chloramphenicol	Phenicol	2	8
Clindamycin	Macrolides, lincosamides	0.5	4
Erythromycin	Macrolides, lincosamides	<0.0625	4
Gentamicin	Aminoglycosides	0.5	4
Kanamycin	Aminoglycoside	2	8
Streptomycin	Aminoglycoside	125	8
Oxytetracycline	Tetracycline	8	8
Vancomycin	Glycopeptide	0.25	4

## Data Availability

Data not presented within the article or Supplementary Materials is available upon request from the corresponding author. The data are not publicly available due to privacy.

## Supplementary Materials

The following supporting information can be downloaded at: https://www.mdpi.com/article/10.3390/microorganisms10051038/s1, Supplemental Table S1: Summary of BLASTn pairwise alignments between BS50 and 20 other *Bacillus* genomes; Supplemental Table S2: Summary of virtual PCR results for the absence of *Bacillus* toxins in BS50.
